# An Improved Method for P2X7R Antagonist Screening

**DOI:** 10.1371/journal.pone.0123089

**Published:** 2015-05-19

**Authors:** Rômulo José Soares-Bezerra, Natiele Carla da Silva Ferreira, Anael Viana Pinto Alberto, André Gustavo Bonavita, Antônio Augusto Fidalgo-Neto, Andrea Surrage Calheiros, Valber da Silva Frutuoso, Luiz Anastacio Alves

**Affiliations:** 1 Laboratory of Cellular Communication, Oswaldo Cruz Institute, FIOCRUZ, Rio de Janeiro, Brazil; 2 Laboratory of Integrated Research, Campus UFRJ-Macaé Professor Aloísio Teixeira, UFRJ, Rio de Janeiro, Brazil; 3 Laboratory of Immunopharmacology, Oswaldo Cruz Institute, FIOCRUZ, Rio de Janeiro, Brazil; Universidad Autonoma de San Luis Potosi, MEXICO

## Abstract

ATP physiologically activates the P2X7 receptor (P2X7R), a member of the P2X ionotropic receptor family. When activated by high concentrations of ATP (i.e., at inflammation sites), this receptor is capable of forming a pore that allows molecules of up to 900 Da to pass through. This receptor is upregulated in several diseases, particularly leukemia, rheumatoid arthritis and Alzheimer's disease. A selective antagonist of this receptor could be useful in the treatment of P2X7R activation-related diseases. In the present study, we have evaluated several parameters using in vitro protocols to validate a high-throughput screening (HTS) method to identify P2X7R antagonists. We generated dose-response curves to determine the EC_50_ value of the known agonist ATP and the IC_s50_ values for the known antagonists Brilliant Blue G (BBG) and oxidized ATP (OATP). The values obtained were consistent with those found in the literature (0.7 ± 0.07 mM, 1.3-2.6 mM and 173-285 μM for ATP, BBG and OATP, respectively). The Z-factor, an important statistical tool that can be used to validate the robustness and suitability of an HTS assay, was 0.635 for PI uptake and 0.867 for LY uptake. No inter-operator variation was observed, and the results obtained using our improved method were reproducible. Our data indicate that our assay is suitable for the selective and reliable evaluation of P2X7 activity in multiwell plates using spectrophotometry-based methodology. This method might improve the high-throughput screening of conventional chemical or natural product libraries for possible candidate P2X7R antagonist or agonist

## Introduction

### Drug Discovery

Herbal medicines have been used worldwide for millennia to treat many diseases. Just over 200 years ago, the first pharmacologically active compound, morphine, was isolated from a plant; since then, it has been used as a potent analgesic. It has been estimated that more than 400,000 plant species exist. Plants have formed the basis of therapeutic medical interventions throughout human history. Despite the availability of modern technology, plants are commonly used to treat various ailments and continue to serve as the basis for many pharmaceutical products [1]. The development of drugs to treat human diseases has been based mainly on a variety of plant-derived compounds. Well-known examples include aspirin, morphine, reserpine, digitalis and quinine. We observed an era which drugs could be isolated, purified, studied and administered in precise doses. Pharmaceutical research expanded after the 1940s, and approximately 80% of drugs developed since then have been derived from natural sources and their analogs or identified through micro-organism screening [2]. In contrast, the focus of drug discovery efforts has shifted from natural products to synthetic compounds in recent decades [3]. Although the expansion of synthetic medicinal chemistry in the 1990s resulted in a decrease in the proportion of new drugs obtained directly from natural products, some synthetic drugs continue to be based on molecules obtained from natural compounds.

Structure-Activity Relationship (SAR) studies, combinatorial chemistry and molecular modeling based on receptor structure are other means by which molecules with selective agonistic or antagonistic activity toward cellular targets can be rationally obtained and synthesized [4].

A wide variety of screening methods can be used for drug discovery; however, only some of these methods are inexpensive, rapid and efficient. Therefore, the approval of new molecular entities has decreased over the past 15 years [5]. An increase in successful drug discovery, especially for drugs targeting difficult chronic targets, is clearly desirable. Thus, it is important to improve drug discovery screening methods.

### P2X7R antagonists as promising therapeutic tools

The roles of ATP and its receptors in physiological and pathophysiological processes have been recognized [6].

Purines and pyrimidines are widely distributed in organisms, where they can act as signaling mediators, promoting various biological effects through purinergic receptors (P1 and P2 receptors). P2 receptors are divided into two families: P2Y and P2X. P2Y receptors (P2YR) are G protein-coupled receptors, whereas P2X receptors (P2XR) are ionotropic receptors [7].

Seven mammalian P2XR subtypes (P2X1-7) have been cloned [8]. P2X7R is expressed in many physiological systems, including the endocrine, cardiovascular, immune, nervous, respiratory, reproductive and digestive systems, as well as in muscular tissues. In the immune system, P2X7R is expressed in several cell types, including macrophages, monocytes, dendritic cells, lymphocytes and mast cells [8].

When activated, P2XR allows for the passage of ions based on an electrochemical gradient. P2X7R is unique in its ability to act as either a low conductance channel (approximately 10 pS) or as a nonselective pore with high conductance (approximately 400 pS). The open pore conformation allows the passage of molecules of up to 900 Da [8]. Based on this property, fluorescent dyes are used in functional assays that evaluate the activation of this receptor; such as ethidium bromide (EB) (MW: 394.31 Da), Lucifer Yellow (LY) (MW: 457.24 Da), YO-PRO-1 (MW: 629 Da) and propidium iodide (PI) (MW: 668.39 Da). Several authors have reported that the formation of a large conductance channel following P2X7R activation is related to the interaction of this receptor with the Pannexin-1 channel; to date, this finding remains controversial [9]. A recently published study by our group found no correlation between the Pannexin-1 and the large conductance channel associated to P2X7R in macrophages [10].

P2X7R plays a crucial role in many physiological processes, including activation of the inflammatory response during pain transduction [11, 12] as well as apoptotic and necrotic processes. Moreover, P2X7R participation has been suggested to play a role in several pathologies, including acute leukemia in children [13], neurodegenerative [14] and parasitic diseases [15] and renal diseases, such as glomerulonephritis [16]. Additionally, upregulation of this receptor has been observed in tumor cells [17, 18].

Due to the findings outlined above, these nucleotide receptors are attracting attention for use in the development of drugs for the treatment of a number of disorders [19–23]. In addition, purinergic receptors have received increasing attention as potential therapeutic targets [24]. The pharmaceutical industry has made a significant investment in researching novel, selective and potent antagonists for these receptors that might be useful as therapeutic agents. Existing antagonists of these receptors are not fully specific, acting on several different cellular targets or on both P2R subtypes [8].

Several fluorometric methodologies that can be used to screen for molecules with P2X7R antagonistic activity have been described in the literature [25–28]. For example, Baxter *et al*. (2003) described the use of a 96-well plate and ethidium bromide in the context of an HTS assay to identify antagonistic compounds, but no essential information regarding the evaluation of the methodology used was published [25]. In the same year, Alcaraz *et al*. described a method using a white 96-well plate and ethidium bromide, in which the analyses were performed using a plate fluorimeter [26]. However, the protocol required a 90-minute incubation at room temperature, and cell viability, possible fluorimetric interference between the white wells and ethidium bromide, and the standardization of this method were not discussed. Another study published in 2009 by Michel *et al*. described an HTS method using a 96-well plate fluorescence reader (FlexStation, Molecular Devices, Sunnyvale, California, United States); the protocol involved treating cells with antagonists for 40 minutes. Prior to the agonist or EB applications, all treatments were performed at room temperature; however, the mean time of incubation after the treatment, the cell viability, the doses of the agonists or antagonists used and the accuracy and reproducibility of this method were not described [28]. Recently, Namovic *et al*. (2012) described a functional HTS assay evaluating P2X7R-induced pore formation using a fluorescence imaging plate reader (FLIPR) and an expensive automated platform, which allowed YO-PRO-1 uptake to be detected in the presence of P2X7R-specific agonists and antagonists in different species. However, the uptake of other dyes (e.g., anionic dyes) and agonist molecules was not tested, nor was possible interference of the Pannexin-1 channel on dye permeation through a P2X7R-induced pore [27].

The purpose of the present study was to develop a fast and low-cost protocol for the identification of natural compounds with potential P2X7R antagonistic or agonistic effects. In this meaning, we used a spectrophotometric microplate reader, which allowed us to simultaneously test between 96 and 386 compounds. We took advantage of the ability of this receptor to open a non-selective pore that promotes the uptake of fluorescent dyes under stimulation with high doses of ATP (> 100 °M). We used J774.G8 cells that natively express P2X7R (10) to evaluate the ATP-dependent dose-response (EC_50_) and the inhibitory concentration (IC_50_) of Brilliant Blue G (a reversible blocker). To verify that dye uptake was occurring through the Pannexin-1 channel, we assessed an experimental protocol using a Pannexin-1 antagonist (mefloquine) and found no correlation between the pore activity of P2X7R and Pannexin-1 channel activity, as previously reported [10]. Thus, we developed a method that has high sensitivity and reproducibility and low cost and may be useful for testing many compounds simultaneously.

## Experimental Section

### Reagents

ATP (adenosine 5'-triphosphate), Brilliant Blue G (BBG), PI, Triton X-100, Oxidized ATP (OATP), HEPES, NaCl, KCl, MgCl_2_, CaCl_2_, Mefloquine (MFQ), Carbenoxolone (CBX) and RPMI 1640 were purchased from Sigma Chemical Co., St. Louis, MO (USA). Fetal bovine serum (FBS) was obtained from Gibco BRL (USA). AZ11645373 (AZ) was obtained from Tocris Bioscience (United Kingdom).

### Cells

The J774.G8 macrophage cell line, which was previously demonstrated by us to natively express P2X7R and the Pannexin-1 channel [10], as well as other P2 subtypes, including P2X4R and P2Y1, P2Y2 and P2Y4 receptors [29]. And U937 human monocyte cell line [30, 31], were routinely maintained in RPMI 1640 medium supplemented with 10% fetal bovine serum at 37°C under an atmosphere containing 5% CO_2_. Every three days, the medium was changed, and the cells were adjusted to a density of 4x10^6^ cells per 150 cm^2^ cell culture flask (Corning). Prior to evaluating the method that was improved upon in this study, cell viability was assessed using Trypan Blue exclusion. Once the range of viability exceeded 90%, the spectrophotometric assay was conducted.

### Spectrophotometric measurement of P2X7R pore activity

Our experimental approach consisted of the measurement of PI or LY uptake by J774.G8 cells through the P2X7R-associated pore. J774.G8 cells (4x10^5^/well) were plated in opaque 96-well plates (Corning) and maintained in culture with RPMI 1640 medium supplemented with 10% fetal bovine serum at 37°C under an atmosphere containing 5% CO_2_ for 24 h. After this time, the medium was exchanged for an extracellular saline solution (in mM): 150 NaCl, 5 KCl, 1 MgCl_2_, 1 CaCl_2_ and 10 HEPES, pH 7.4. The following protocol was then performed: the cells were stimulated with ATP [5 mM] for 15 minutes at 37°C under an atmosphere containing 5% CO_2_. PI [50 nM] or LY [3 mM] was then added to the cell cultures for 5 minutes (to avoid the uptake of these dyes via other mechanisms, such as pinocytosis) in the treatments using LY. After this incubation, the saline solution was exchanged to eliminate interference from non-permeated dye. The plates were then assessed using a spectrophotometer (SpectraMax M5, Molecular Devices, Sunnyvale, California, United States); PI uptake was measured by excitation at 488 nm -λ and emission at 590 nm λ, whereas LY uptake was measured by excitation at 485 nm -λ and emission at 528 nm λ. Three wells were used for each control group: the positive control group was treated with Triton X-100 [0.1%] (which was directly added to the control wells), the negative control group was treated only with extracellular saline solution, and the remaining groups were treated with ATP [5 mM] following exposure to dye (PI or LY) for longer than 5 minutes.

### Drug Treatments

The protocols including treatment with the P2X7 antagonist (the competitive antagonist, BBG [0.048–100 μM]) or the irreversible antagonist (oxidized ATP (OATP), [0.048–400 μM]) were the same as those described above. Three wells were used to test each concentration. BBG-treated wells were incubated for 15 minutes at 37°C under an atmosphere containing 5% CO_2_, whereas OATP-treated wells were incubated for 60 minutes at 37°C under an atmosphere containing 5% CO_2_. After both treatments, ATP stimulation was applied for 15 minutes; then, PI or LY was added for an additional 5 minutes before spectrophotometric analysis.

### Imaging analysis

In addition to the spectrophotometric analyses, we also performed fluorescence microscopy analyses (Nikon Eclipse TE2000S) as previously described to verify dye uptake in ATP-activated cells. Images were captured using a Nikon digital camera system.

### Lactate Dehydrogenase (LDH) detection assay

The supernatants of J774.G8 cells that had been previously treated for 15 minutes with five different concentrations of ATP were performed according to the instructions included in the LDH detection kit, which was purchased from Doles (Goiania, GO, Brazil).

### Cytotoxicity assay using the MTT technique

J774.G8 cells (4x10^5^ cells/well) were treated for 15 minutes with five different concentrations of ATP, after which point, the medium was changed and 3-[4,5-dimethylthiazol-2-yl]-2,5-diphenyl-tetrazolium bromide (MTT) was added. After incubation for 3 h at 37°C, the reactions were interrupted with DMSO, and the absorbance was measured using a spectrophotometer (SpectraMax M5, Molecular Devices, Sunnyvale, California, United States) at 450 nm.

### Pannexin-1 Blockage Assay

The same spectrophotometric protocol previously described was assessed, including the same controls, using both J774.G8 and U937 cells, plus cell treatment with the Pannexin-1 antagonists—the Mefloquine [1 μM] and Carbenoxolone [100 μM]. In this experimental protocol, we also assessed the specific blockage of P2X7R by its selective antagonist, AZ11645373, in a specific concentration for each cell type: [10 μM] for U937 and [100 μM] for J774.G8 cells. Three well were used for its concentration and incubated for 15 min at 37°C and an atmosphere of 5% of CO_2_. The treatments occurred before ATP stimulation for 15 min then the PI was added for more 5 min before the spectrophotometric analysis.

### Data analysis

Each sample was measured in triplicate and all experiments were repeated a minimum of three times by each operator. In the present study, two operators participated in all of the experimental steps. The fit was evaluated using the R^2^ coefficient [32]. The data are expressed as the means ± standard deviation. To determine whether the samples followed a Gaussian distribution, we used two normality tests: D’Agostino's K-squared test and Pearson's chi-squared test. When the data followed a Gaussian distribution, they were compared using one-way ANOVA, followed by Tukey's post-hoc analysis. Data that did not follow a Gaussian distribution were compared using the Kruskal-Wallis, followed by Dunns post-hoc analysis. *P* values of 0.05 or less were considered significant. The tests used are specified in the figure legends and were two-tailed and paired. We also assessed the quality of our improved HTS assay by calculating the Z-factor, which is a valuable tool that can be used to evaluate the robustness and suitability of HTS assays and that can be useful for assay optimization and validation [33]. This parameter is calculated using the following equation:

$$\text{Z}\;=\;1\;-\;\frac{\text{3SD}\;\text{of}\;\text{sample}\;\text{+}\;3\text{SD}\;\text{of}\;\text{control}}{\text{mean}\;\text{of}\;\text{sample}\;\text{-}\;\text{mean}\;\text{of}\;\text{control}}$$

The SD values represent the standard deviation values (of the control or sample). We calculated the Z-factor using ATP as a control, and the means of the samples were calculated using the dye uptake assay values for PI or LY, accordingly.

## Results and Discussion

### Effects of ATP on PI uptake in J774.G8 cells

**Fig 1:**
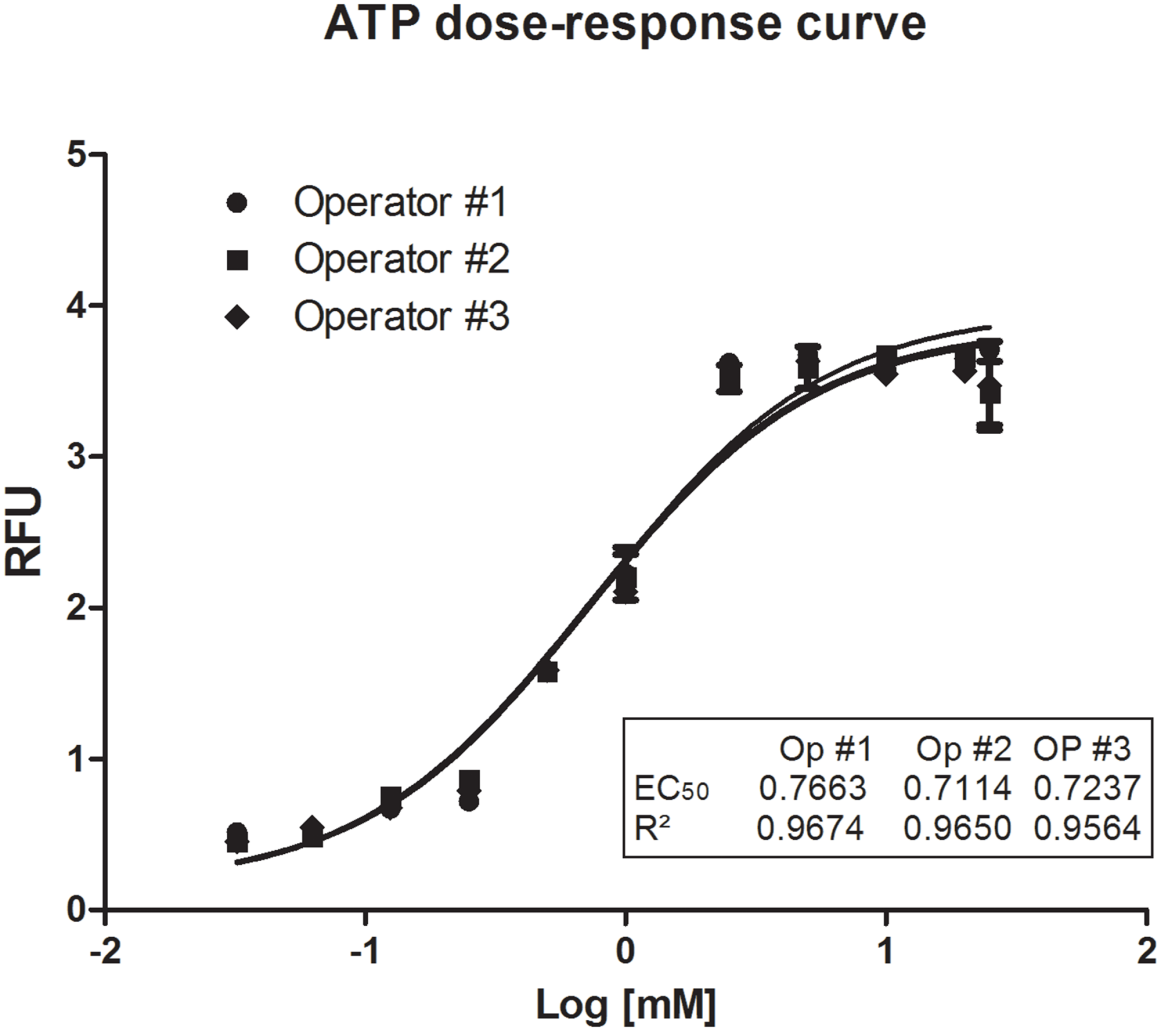
ATP dose-response curve. J774.G8 cells were plated in opaque 96-well plates and treated with various concentrations of ATP from 0.321 to 25 mM in combination with PI [50 nM]. The data represent the means ± S.D. of three independent experiments in triplicate. The results were analyzed using the Kruskal-Wallis, followed by Dunns post-hoc analysis; * p<0.05.

To determine whether our screening method is reliable and reproducible, we established a dose-response curve using ATP. We plated cells in opaque 96-well plates and added various concentrations of ATP from 0.321 to 25 mM. After 15 minutes, we added PI and measured the absorbance using a spectrophotometer. The calculated EC_50_ of ATP using our method was 0.7 ± 0.07 mM. The dose-response curve for ATP using three different operators is shown in Fig 1. This value of EC_50_ was similar to that described in the literature for the activation of selective dye permeation pathways by P2X7R [34]. Our method resulted in a low EC_50_ value compared to values obtained using other methodologies described in the literature (Table 1), confirming the high accuracy of this method. Inter- and intra-operator mensurements did not differ significantly, and the mean difference between the daily measurements made by the two operators was 0.963 (p<0.05) (Fig 1).

**Table 1:** IC_50_ or EC_50_ values for P2X7R agonists or antagonists using different methods.

	ATP (EC_50_)	BBG (IC_50_)*	OATP (IC_50_)	Reference
Cytometry	2–4 mM	N.D.**	300 uM	Coddou *et al*. 2011
Electrophysiology	2–4 mM	N.D.**	300 uM	Coddou *et al*. 2011
FLIPR	N.D.**	6.71± 0.05 uM	N.D.**	Namovic *et al*. 2012
Spectrophotometry	0.7 ± 0.07 mM	1.3–2.6 μM	173–285 uM	Present study

*IC_50_ for natively expressed mP2X7R in J774.G8 cells.

**N.D. = Not determined

### Antagonist-mediated inhibition of the P2X7 receptor

We next determined whether the main P2X7R competitive antagonist, BBG, could block the pore associated with the receptor in a dose-dependent manner and with a similar IC_50_ value. Brilliant Blue G (BBG) is an analog of FD&C blue dye, a widely used food additive that is commonly used as a selective P2X7R antagonist [35]. Some authors have suggested that the low toxicity and high selectivity of BBG make this compound useful for blocking the potential adverse effects of P2X7R activation in several pathological conditions [35].

**Fig 2:**
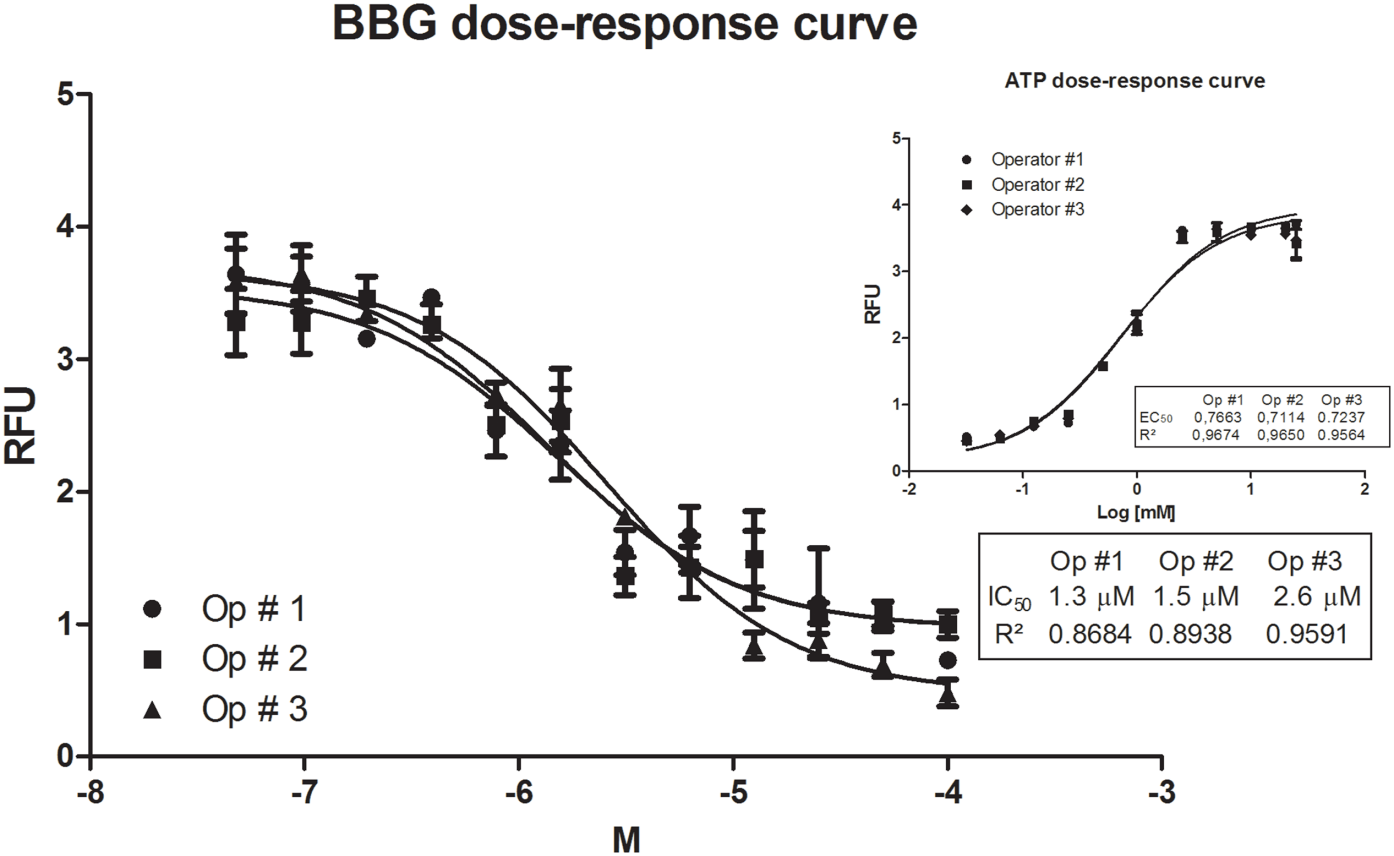
BBG dose-response curve. J774.G8 cells were plated in opaque 96-well plates and treated with various concentrations of BBG from 0.048 to 100 μM in combination with PI [50 nM] and ATP [5 mM]. The data represent the means ± S.D. of three independent experiments in triplicate. The results were analyzed using the Kruskal-Wallis, followed by Dunns post-hoc analysis; * p<0.05.

As expected, BBG blocked PI transport, and the IC_50_ value ranged from 1.3 to 2.6 μM (Fig 2). As mentioned previously, the inter- and intra-operator variations in the IC_50_ value were not significant, and the dose-response curves did not differ significantly (Fig 2). An IC_50_ value of 6.71± 0.05 μM for mP2X7R is reported in the literature [27], and we compared this value to our results because our model was based on a mouse macrophage cell line (J774.G8). Using our improved methodology, we obtained IC_50_ values for BBG and mP2X7R that ranged from 1.3 to 2.6 μM. These values are lower than those reported previously, indicating a more sensitive and specific antagonistic action on this receptor.

**Fig 3:**
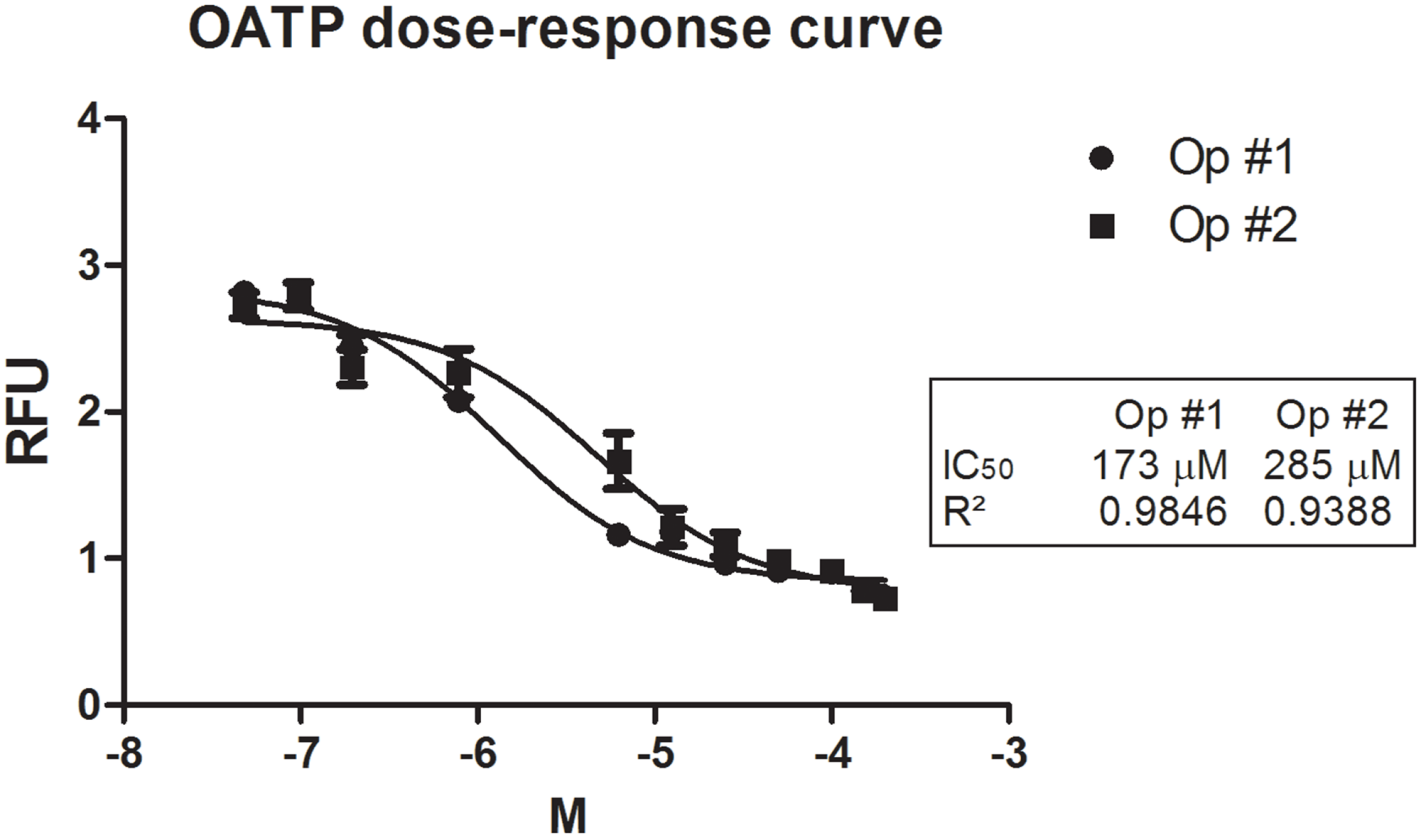
OATP dose-response curve. J774.G8 cells were plated in opaque 96-well plates and treated with various concentrations of OATP from 0.048 to 400 μM. The data represent the means ± S.D. of three independent experiments in triplicate. The results were analyzed using the Kruskal-Wallis, followed by Dunns post-hoc analysis; * p<0.05.

**Fig 4:**
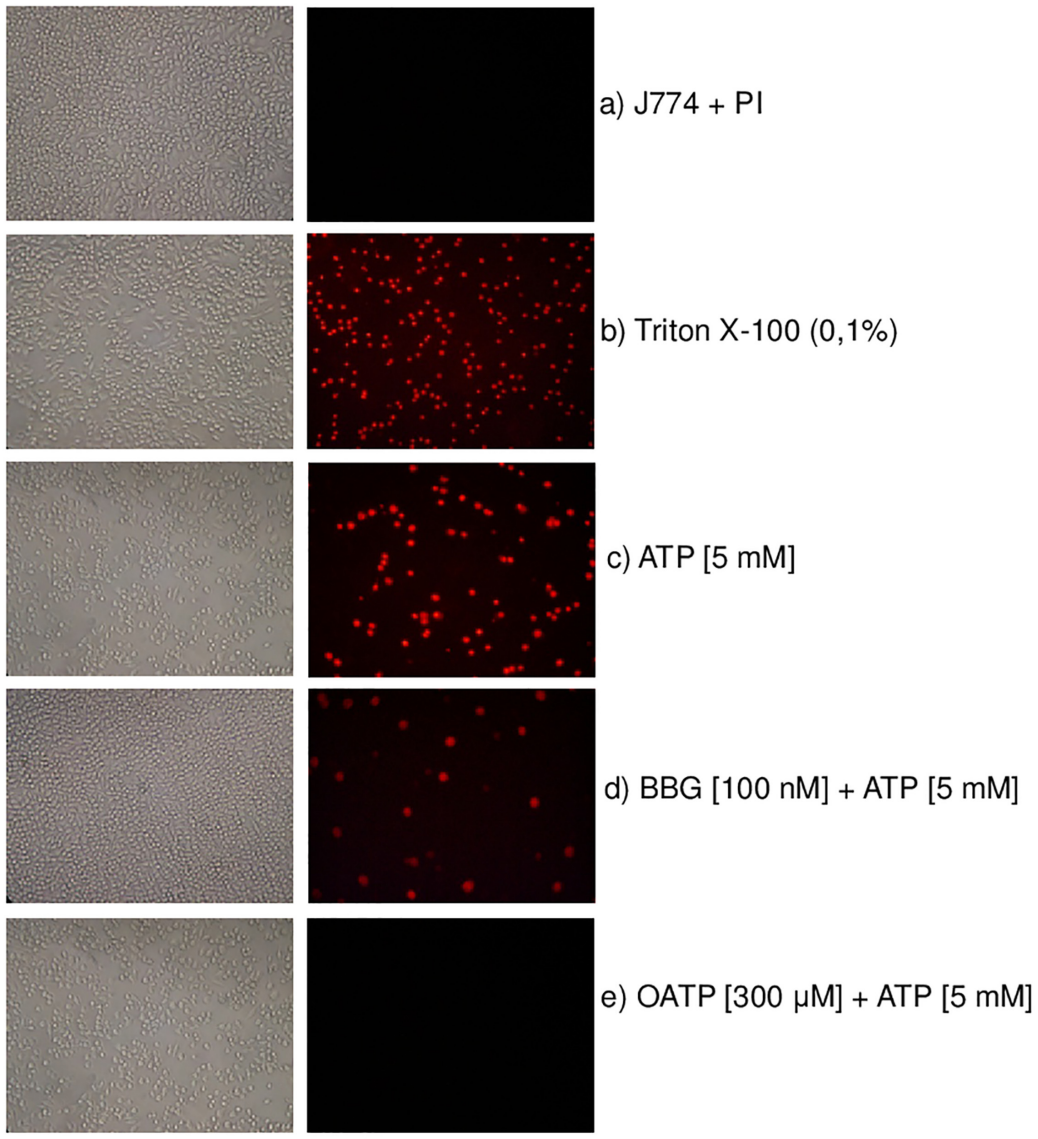
Evaluation of the inhibitory activity of BBG on P2X7 using fluorescence microscopy. J774.G8 cells were treated as follows: (a) Control (J774 plus PI [50 nM]), (b) permeabilization control (Triton-X 100 [0.1%]), (c) control cells plus ATP [5 mM], (d) cells treated with BBG [100 nM] plus ATP [5 mM] and (e) cells treated with OATP [300 μM] plus ATP [5 mM]. The profiles are representative of three to seven independent experiments that were performed in triplicate. The images were captured using a Nikon digital camera system with a total magnification of 43.8X.

After examining the antagonistic activity of BBG on P2X7R using our methodology, we evaluated the activity of other antagonist molecules on this receptor. For this purpose, we used a specific irreversible antagonist, oxidized ATP (OATP). As shown in Fig 3, we established an IC_50_ in a range from 173 to 285 μM and an inhibitory curve for oxidized ATP [0.048–400 μM]; these values are less than those obtained using other methods (Table 1). To confirm the data that we obtained using other methodologies that are currently used to screen molecules with activity at this receptor, we used a dye uptake protocol based on J774.G8 cells treated with known P2X7R agonists and antagonists at specific concentrations that are known to activate only P2X7R [9] prior to conducting fluorescence microscopy analyses (Fig 4). The images confirmed that the P2X7 pore was activated by ATP and that it was blocked by both BBG and OATP. When we compared the IC_50_ and EC_50_ values obtained using this improved methodology with values obtained using other methodologies, our results were more sensitive and accurate than those obtained using methods that are usually used to screen compounds with P2X7R activity (Table 1).

**Fig 5:**
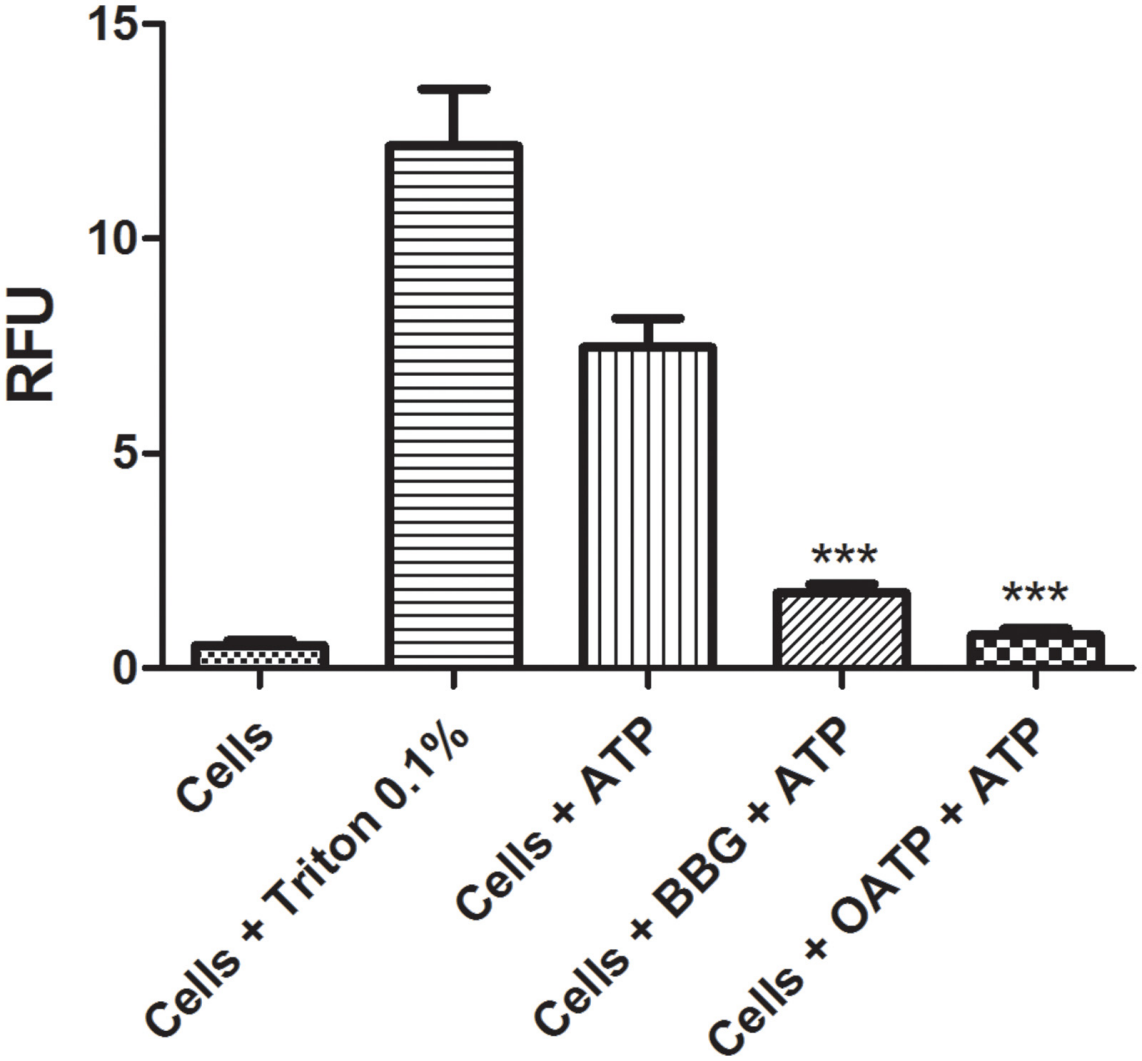
Anionic dye uptake. J774.G8 cells were plated in opaque 96-well plates after the following treatments: a) Cells plus LY [3 mM], b) cells plus ATP [5 mM] plus LY [3 mM], c) cells plus Triton X-100 [0.1%] plus LY [3 mM], d) cells plus BBG [100 nM] plus ATP [5 mM] plus LY [3 mM] and e) cells plus OATP [300 μM] plus ATP [5 mM] plus LY [3 mM]. The data represent the means ± S.D. of three independent experiments in triplicate. The results were analyzed using the Kruskal-Wallis, followed by Dunns post-hoc analysis; * p<0.05.

Some authors have described the existence of two activated pores that are related to P2X7R activation, one of which allows for the passage of cationic molecules and the other of which allows for the uptake of anionic molecules [35]. We conducted an experiment to assess our method of evaluating the inhibition of anionic dye uptake by the P2X7R pore. As shown in Fig 5, we treated J774.G8 cells with ATP [5 mM], the antagonists BBG [100 nM] and OATP [300 μM] and an anionic dye, LY [3 mM]. The results show the inhibitory effects of these antagonists on dye uptake, thereby validating our method of investigating compounds with P2X7R antagonistic activity by inhibiting the uptake of anionic molecules by the activated pore. To confirm the robustness of our HTS assay, we used a statistical parameter termed the Z-factor, which is used to evaluate the suitability of a high-throughput screening assay based on the range of the signal and the internal variation of the equipment. A score of 1>Z≥0.5 indicates a good assay, and a score of Z = 1 indicates a perfect assay. Our calculated Z-factor for the HTS assay was 0.635 using PI and 0.876 using LY [34].

**Fig 6:**
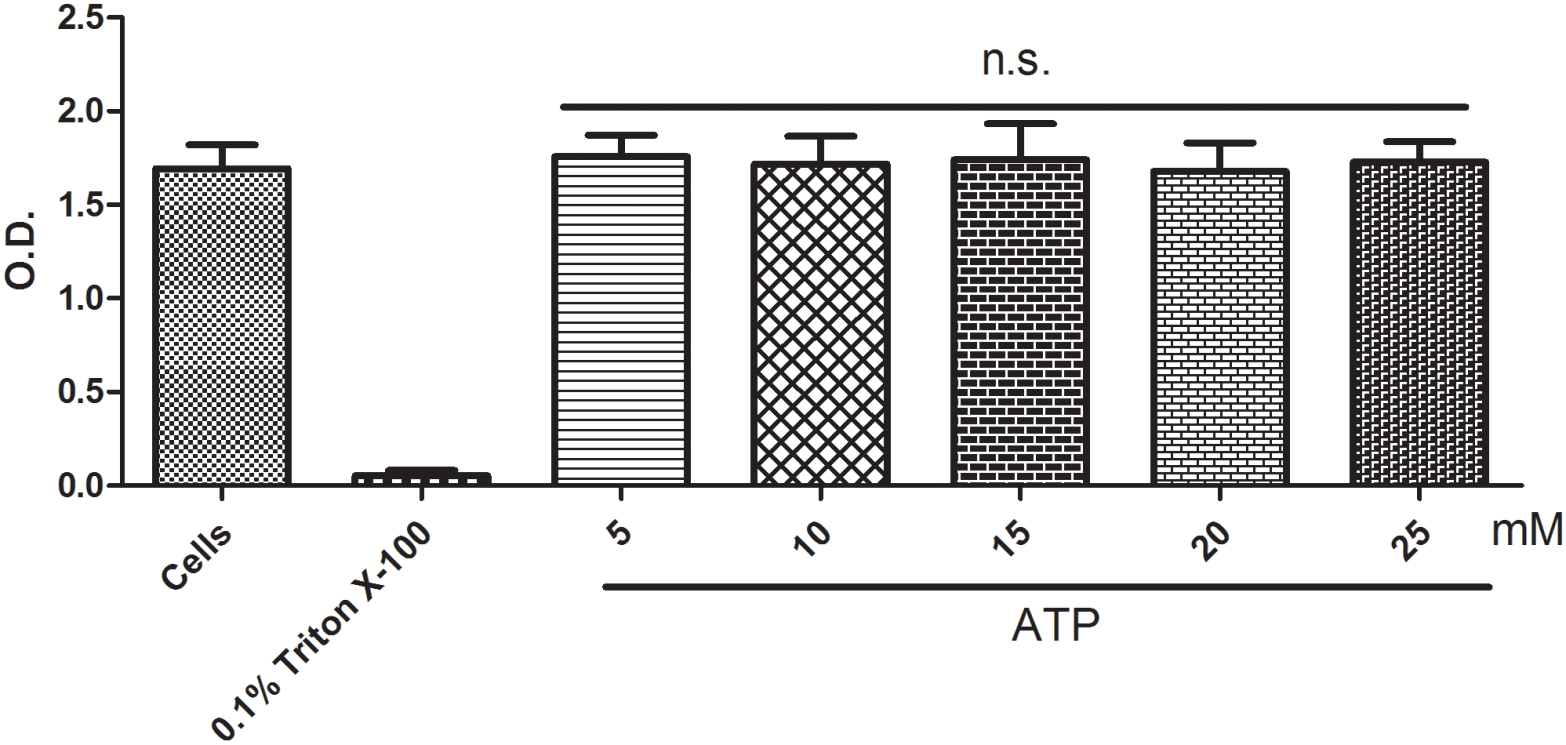
MTT cytotoxicity assay. J774.G8 cells that were treated for 15 minutes with five concentrations of ATP were analyzed. (a) Untreated cells, (b) cells plus Triton X-100 [0.1%], (c) cells plus ATP [5 mM], (d) cells plus ATP [10 mM], (e) cells plus ATP [15 mM], (f) cells plus ATP [20 mM] and (g) cells plus ATP [25 mM]. The data represent the means ± S.D. of three independent experiments in triplicate. The results were analyzed using ANOVA, followed by Tukey's post-hoc test; * p<0.05.

**Fig 7:**
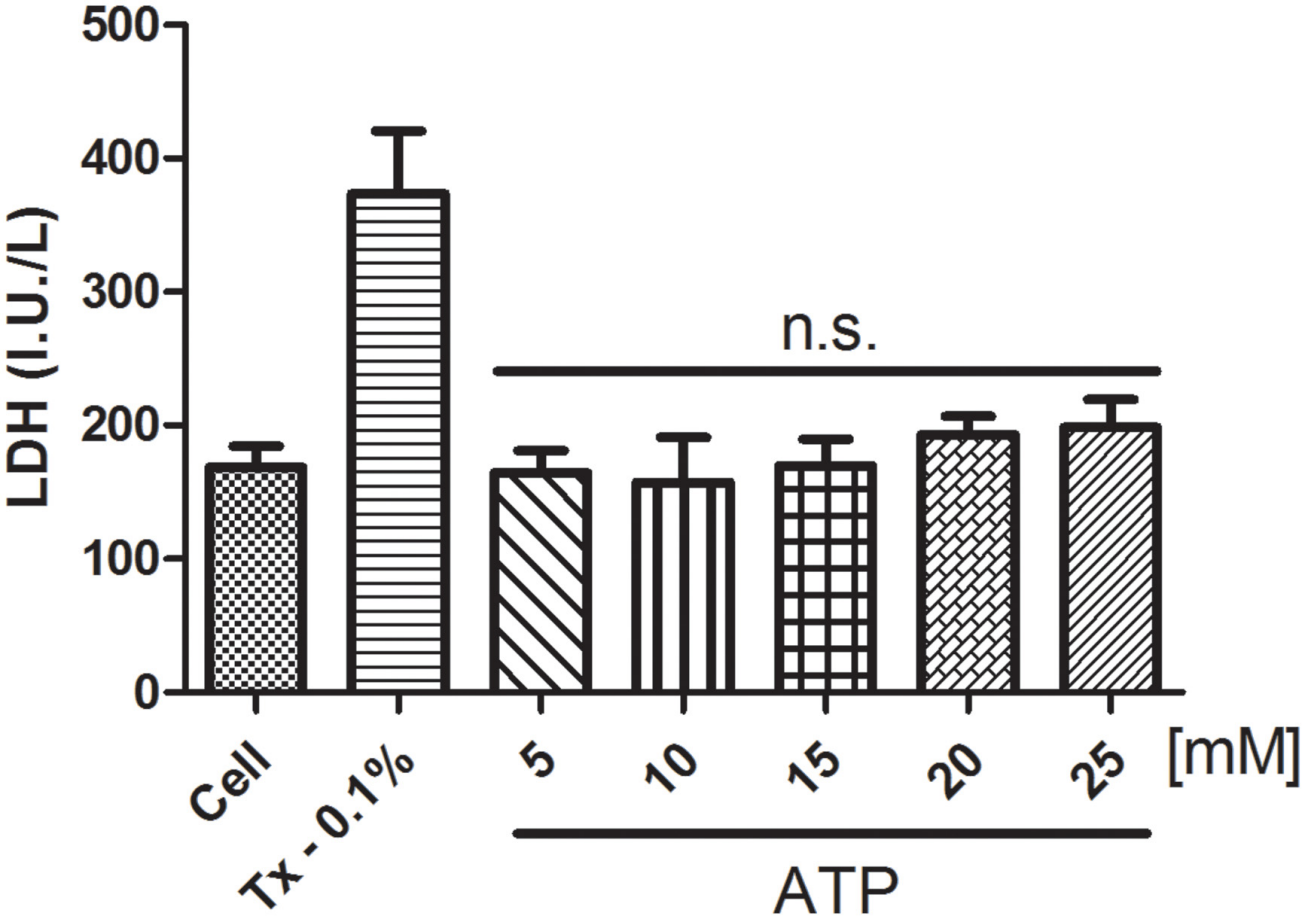
LDH assay in J774.G8 Cells. The supernatants of cells that had been treated for 15 minutes with five concentrations of ATP were analyzed. (a) Untreated cells, (b) cells plus Triton X-100 [0.1%], (c) cells plus ATP [5 mM], (d) cells plus ATP [10 mM], (e) cells plus ATP [15 mM], (f) cells plus ATP [20 mM] and (g) cells plus ATP [25 mM]. The data represent the means ± S.D. of three independent experiments in triplicate. The results were analyzed using ANOVA, followed by Tukey's post-hoc test; * p<0.05.

We also evaluated the cytotoxicity of the ATP treatments at the concentrations used in this work for 15 minutes using the LDH and MTT assays. No toxicity was observed until 1 hour had elapsed (as shown in Figs 6 and 7).

**Fig 8:**
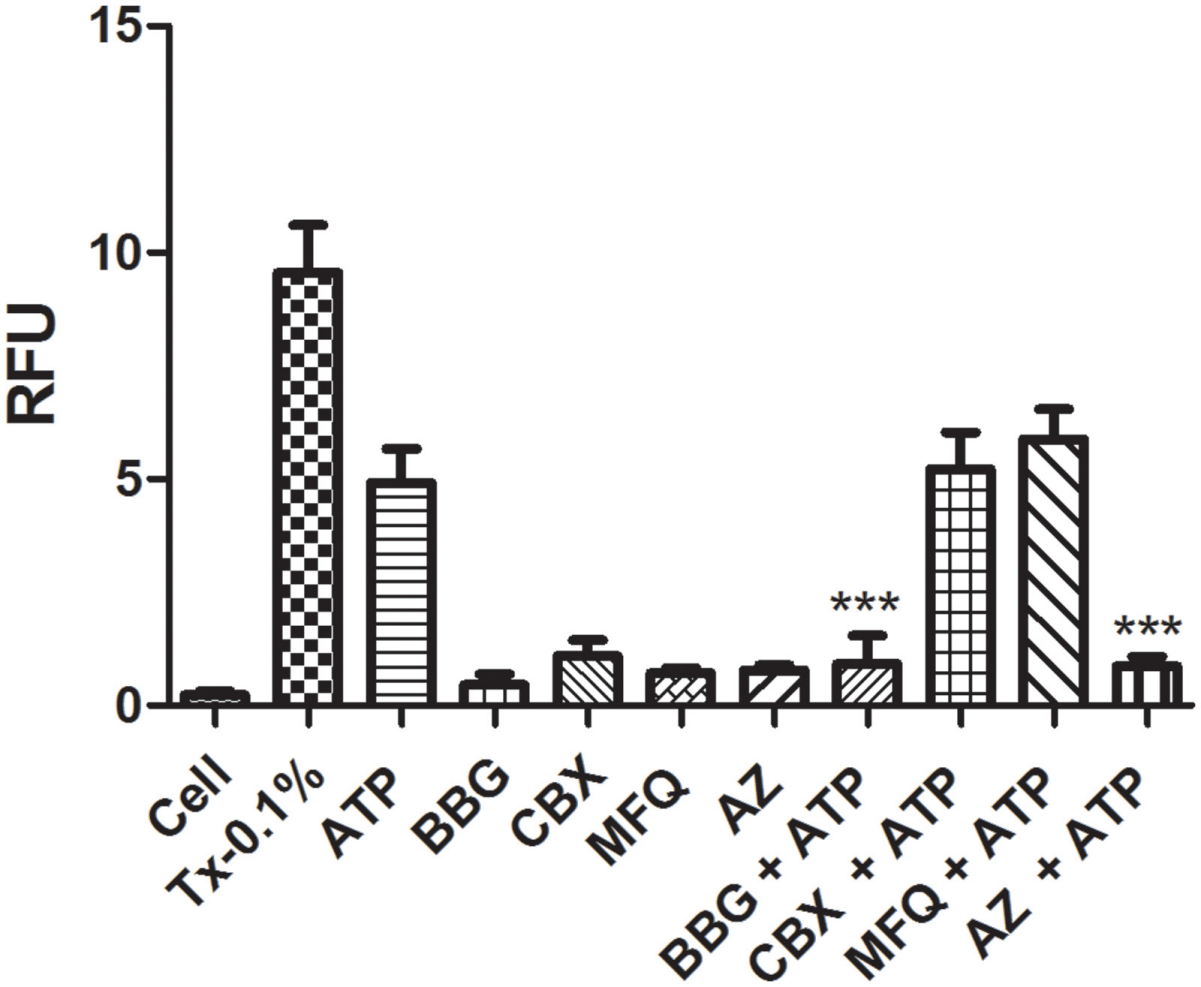
Pannexin-1 Blockage Assay in U937 Cells. U937 cells were plated in opaque 96-well plates following the subsequent treatments: a) Cells plus PI [50 nM]; b) cells plus Triton X-100 [0.1%] plus PI [50 nM]; c) cells plus ATP [5 mM] plus PI [50 nM]; d) cells plus BBG [100 nM] plus PI [50 nM]; e) cells plus Carbenoxolone [100 μM] plus PI [50 nM]; f) cells plus Mefloquine [1 μM] plus PI [50 nM]; g) cells plus AZ11645373 [10 μM] plus PI [50 nM]; h) cells plus BBG [100 nM] plus ATP [5 mM] plus PI [50 nM]; i) cells plus Carbenoxolone [100 μM] plus PI [50 nM] plus ATP [5 mM]; j) Cell plus Mefloquine [1 μM] plus ATP [5 mM] plus PI [50 nM]; l) cells plus AZ11645373 [10 μM] plus PI [50 nM] plus ATP [5 mM]. Date represents mean ± S.D of three independent experiments in triplicate. The result was analyzed using the Kruskal-Wallis, followed by Dunns post-hoc analysis; * p<0.05.

**Fig 9:**
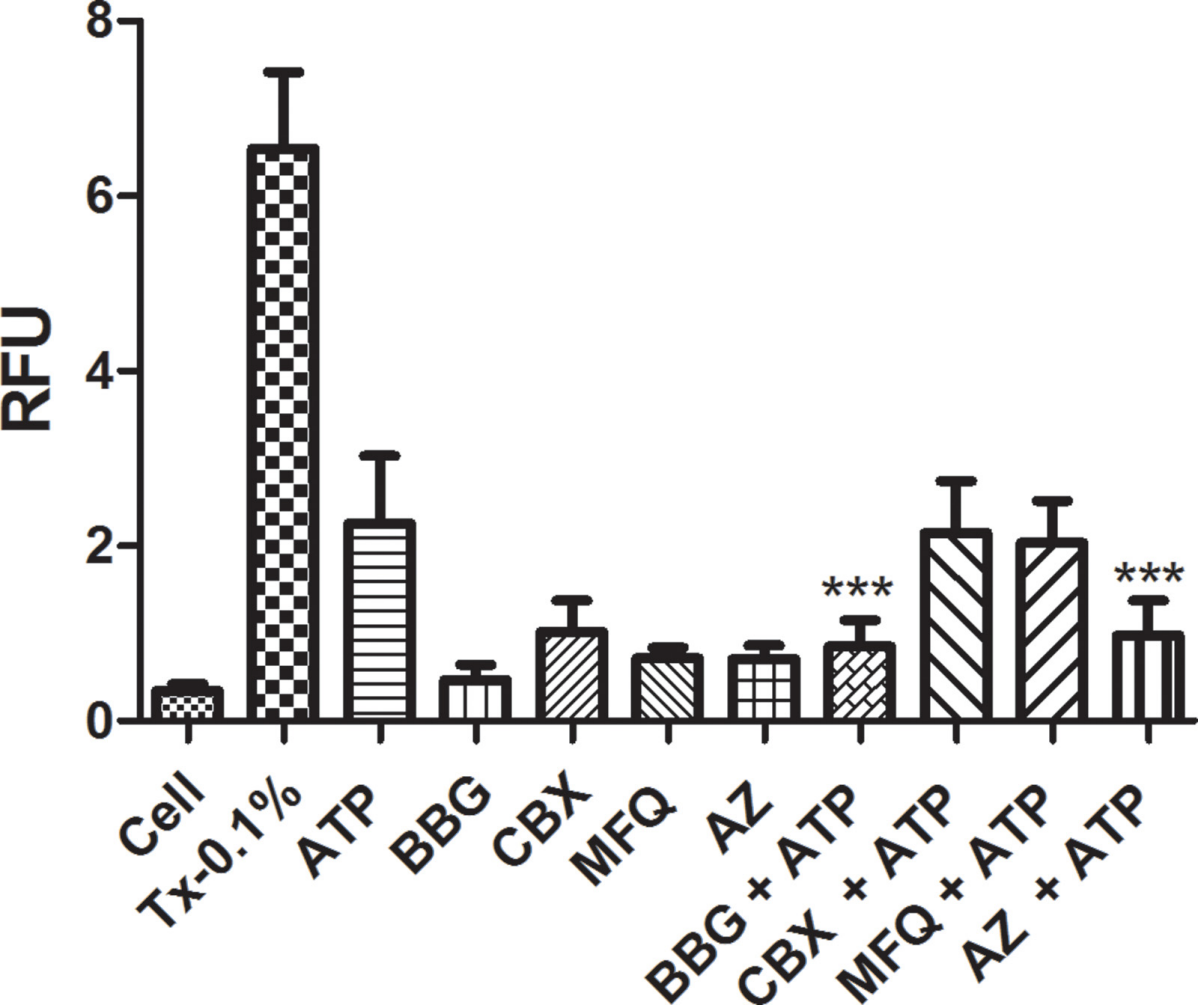
Pannexin-1 Blockage Assay in J774.G8 Cells. J774.G8 cells were plated in opaque 96-well plates following the subsequent treatments: a) Cells plus PI [50 nM]; b) cells plus Triton X-100 [0.1%] plus PI [50 nM]; c) cells plus ATP [5 mM] plus PI [50 nM]; d) cells plus BBG [100 nM] plus PI [50 nM]; e) cells plus Carbenoxolone [100 μM] plus PI [50 nM]; f) cells plus Mefloquine [1 μM] plus PI [50 nM]; g) cells plus AZ11645373 [100 μM] plus PI [50 nM]; h) cells plus BBG [100 nM] plus ATP [5 mM] plus PI [50 nM]; i) cells plus Carbenoxolone [100 μM] plus PI [50 nM] plus ATP [5 mM]; j) cells plus Mefloquine [1 μM] plus ATP [5 mM] plus PI [50 nM]; l) cells plus AZ11645373 [100 μM] plus PI [50 nM] plus ATP [5 mM]. Date represents mean ± S.D of three independent experiments in triplicate. The result was analyzed using the Kruskal-Wallis, followed by Dunns post-hoc analysis; * p<0.05.

Pannexin-1 has been implicated as the protein associated with the P2X7R pore [30,36]. For this reason, we examined whether our methodology was specific for P2X7R pore activity. As seen in Figs 8 and 9, the treatments with two different Pannexin-1 antagonists (mefloquine and cabenoxolone), simultaneously with the P2X7R agonist ATP did not block dye uptake, indicating that this uptake may have occurred via the opened P2X7R pore or other associated pore rather than via the Pannexin-1 channel [37]. Additionally, the treatment with the selective P2X7R antagonist AZ11645373 blocked the dye uptake. These results reinforces the applicability of our standardized methodology to verify the blockage or non-blockage of dye uptake through P2X7R activation only. When taken together, our results validated the improved methodology developed in this study and demonstrated its applicability to P2X7R antagonist screening. P2X7R is primarily expressed in immune cells and cells of hematopoietic origin, such as mast cells and microglia, and has a longer carboxy-terminal tail than the six other members of the P2X receptor family. As mentioned previously, this receptor plays a crucial role in a significant number of pathophysiological conditions.

Pharmaceutical companies have expressed great interest in the development of new medicines, and the rate of patent protection loss is significantly greater than the rate of new drug discovery. Given this consideration, the market for new pharmaceutical compounds is relatively fragile because a small number of products represent a significant portion of the market. For example, eight products accounted for 58% of Pfizer’s annual worldwide sales of $44 billion in 2007 [2]. When such drugs lose their patent protection, the associated sales revenue can decrease by 80%. In addition, the competition from generic drug manufacturers, who do not participate in the drug discovery process, is intense; generic manufacturers produce 67% of all drugs prescribed in the United States [2].

Many methodologies are used to screen currently available drugs. However, some features, such as the speed of execution and reliability, are critical. Furthermore, it is important that these screening methods are able to test a large number of candidates. Currently, high-throughput screening (HTS) programs in drug discovery rely mainly on combinatorial chemistry libraries. When compared to the very large number of natural products, combinatorial chemistry libraries are relatively limited with respect to molecular diversity. Our improved methodology might possess great advantages over currently available methodologies because it can easily be used to screen a wide spectrum of compounds of synthetic or natural origin.

## Conclusions

Synthetic compounds are currently being tested and patented by the pharmaceutical industry as potential antagonists for clinical use [38]. There is a strong clinical and industrial interest in specific P2X7R antagonists for use in the treatment of many diseases, such as those related to inflammation or pain, as well as cancer. Advances in the selection and development of new P2 receptor antagonists will certainly be relevant and it is of significant technological interest. To facilitate the speed of the discovery of potential antagonists, the development of a simple, rapid and relatively inexpensive method for testing compounds on a large scale is necessary. However, some limitations have to be considered for standarzing a HTS method to discover novel compounds with possible activity on the P2X7R. Such limitations concern the existence of polymorphisms and splice variants of this receptor in some cell types, which it may influence the analysis of the obtained data [39]. A way to minimize these limitations is based on the selection of the cell type to be used during the standardization process. In the present study, an improved method was standardized and tested using known P2X7R antagonists, generating results that validated this protocol; this method is applicable to the selection of new compounds with P2X7R inhibitory activity.
