# Correction: An Improved Method for P2X7R Antagonist Screening

**DOI:** 10.1371/journal.pone.0137488

**Published:** 2015-08-31

**Authors:** Rômulo José Soares-Bezerra, Natiele Carla da Silva Ferreira, Anael Viana Pinto Alberto, André Gustavo Bonavita, Antônio Augusto Fidalgo-Neto, Andrea Surrage Calheiros, Valber da Silva Frutuoso, Luiz Anastacio Alves

There are errors in the seventh sentence of the Abstract. Specifically, the values provided for ATP, BBG and OATP are incorrect. The correct sentence is: The values obtained were consistent with those found in the literature (0.7 ± 0.07 mM, 1.3–2.6 μM and 173–285 μM for ATP, BBG and OATP, respectively).

Additionally, there are errors in the units of Table 1. The correct units for columns BBG (IC_50_) and OATP (IC_50_) are μM. Please see the corrected Table 1 here.

**Table 1:** IC_50_ or EC_50_ values for P2X7R agonists or antagonists using different methods.

	ATP (EC_50_)	BBG (IC_50_)*	OATP (IC_50_)	Reference
Cytometry	2–4 mM	N.D.**	300 μM	Coddou *et al*. 2011
Electrophysiology	2–4 mM	N.D.***	300 μM	Coddou *et al*. 2011
FLIPR	N.D.**	6.71±0.05 μM	N.D.**	Namovic *et al*. 2012
Spectrophotometry	0.7 ±0.07 mM	1.3–2.6 μM	173–285 μM	Present study

*IC_50_ for natively expressed mP2X7R in J774.G8 cells.

**N.D. = Not determined

## References

[1] Soares-Bezerra RJ, Ferreira NCdS, Alberto AVP, Bonavita AG, Fidalgo-Neto AA, Calheiros AS, et al (2015) An Improved Method for P2X7R Antagonist Screening. PLoS ONE 10(5): e0123089 doi:10.1371/journal.pone.0123089 25993132 10.1371/journal.pone.0123089 PMC4437783

